# Chinese herbal compound prescription for Endometriosis: A protocol for systematic review and meta-analysis

**DOI:** 10.1097/MD.0000000000022698

**Published:** 2020-10-16

**Authors:** Xiusong Tang, Jing Chen, Peiqi Ou, Jingqin Chen, Shaohang Lan, Jiajing Luo, Yehao Luo, Yuzhi Shang, Gang Fang

**Affiliations:** Guangxi university of Traditional Chinese Medicine, Nanning, Guangxi Province, China.

**Keywords:** Chinese herbal compound prescription, endometriosis, meta-analysis, protocol, systematic review

## Abstract

**Background:**

Endometriosis (EMT) is one of the common diseases of women of childbearing age. EMT destroys the anatomical structure of the pelvis, which leads to abnormal ovulation and endocrine abnormalities. It also affects embryo implantation and makes patients infertile. Recently, it is confirmed that Chinese medicine also have an excellent clinical efficacy on EMT. Compared with the conventional western medicine treatment, it effectively relieve pain and other concomitant symptoms.

**Methods and analysis:**

The following databases will be searched for relevant information before July 2020: PubMed, Embase, Cochrane Library, Web of Science, and CNKI. Major results: the overall effective rate, VAS score. Secondary outcomes: blood serum estradiol (E2), progesterone (P), Follicle-Stimulating Hormone (FSH), adverse events. Data will be collected independently by 2 researchers, and the risk of bias in meta-analysis will be evaluated according to “Cochrane Handbook for Systematic Reviews of Interventions”. All data analysis will be conducted using Review Manager V.5.3. and Stata V.12.0.

**Results:**

The curative effect and safety of Chinese herbal compound prescription treatment for EMT patients will be evaluated systematically.

**Conclusion:**

The systematic review of this study will summarize the currently published evidence of Chinese herbal compound prescription treatment for EMT to further guide its promotion and application.

**Ethics and dissemination:**

The private information from individuals will not be published. This systematic review also will not involve endangering participant rights. Ethical approval is not required. The results may be published in a peer-reviewed journal or disseminated in relevant conferences.

**Open Science Framework (OSF) registration number:**

https://osf.io/p5nrk.

## 1 Introduction

Endometriosis (EMT) is a common gynecological disease, which mainly related to chronic inflammatory hormone dependence, and eventually develops into chronic pelvic pain and infertility. The growth of ectopic EMT is one of the main characteristics.^[1]^ Its clinical incidence is about 10%.^[2]^ At present, researches have shown that the occurrence of EMT are closely related to environmental factors, genetic factors, oxidative and nitrative stress response, inflammation, angiogenesis, extracellular matrix degradation, and oxidative stress interaction.^[3–5]^ In addition, more and more evidence shows that various epigenetic aberrations may be related to the pathogenesis of EMT, including differential expression of DNA methyltransferase, histone deacetylation, and non-coding microRNA.^[6]^ Clinically, the main methods for the treatment of EMT include surgery, hormone contraceptive, the combination of progesterone and anti-progesterone, GnRH agonists and antagonists, aromatase inhibitors, danazol, and non-steroidal anti-inflammatory drugs (NSAID).^[7]^ In recent years, traditional Chinese medicine has gradually shown its remarkable therapeutic effect in the prevention and treatment of various diseases. For EMT, traditional Chinese medicine improves pelvic pain and the quality of life to promote female reproductive health. However, there is a lack of evidence of results of combination of Chinese and western medicine in treating EMT. Therefore, the paper will evaluate the effectiveness and safety of Chinese herbal compound prescription treatment for EMT. This review will be the first evaluation of the impact of Chinese herbal compound prescription treatment.

## 2 Objectives

In a randomized controlled trial (RCT), the efficacy and side effects of Chinese herbal compound prescription in treating EMT have been evaluated systematically. We expect to provide reference for EMT treatment in the field of traditional Chinese medicine.

## 3 Methods

### 3.1 Study registration

The protocol of the systematic review has been registered.

Registration: OSF Preregisration. 2020, Sep.7. osf.io/p5nrk. This systematic review protocol will be conducted and reported strictly according to Preferred Reporting Items for Systematic Reviews and Meta-Analyses (PRISMA)^[8]^ statement guidelines, and the important protocol amendments will be documented in the full review.

### 3.2 Inclusion and exclusion criteria for study selection

#### 3.2.1 Inclusion criteria

Inclusion criteria are all randomized controlled trials (RCTs), which main treatment of EMT is Chinese herbal compound. The language of the trials to be included only Chinese or English.

#### 3.2.2 Exclusion criteria

Following studies will be excluded.

1. Repeated publications.
2. Review of literature and cases.
3. Animal studies.
4. Incomplete literature.
5. Non-randomized controlled trials.

### 3.3 Types of participants

We will include RCTs of participants with no fertility needs; patients diagnosed with endometriosis and requiring laparoscopic surgery; except for endometriosis, there are no other functional diseases. Exclusion criteria: Exclude patients who are allergic to the drugs used in this study; pregnant women; patients who have taken hormone drugs for nearly 2 months before entering the group.

### 3.4 Interventions and controls

Interventions included treatment with Chinese herbal compound. The control group only received conventional western medicine treatment. The routine treatment of each RCT may not be identical, but the use of Chinese herbal compound is the only difference between intervention and control.

### 3.5 Type of outcome measures

#### 3.5.1 Main outcomes

1. the overall effective rate;
2. VAS score

#### 3.5.2 Additional outcomes

1. blood serum estradiol (E2);
2. progesterone (P);
3. Follicle-Stimulating Hormone (FSH);
4. Adverse events.

### 3.6 Search methods

#### 3.6.1 Search resources

**Figure 1:**
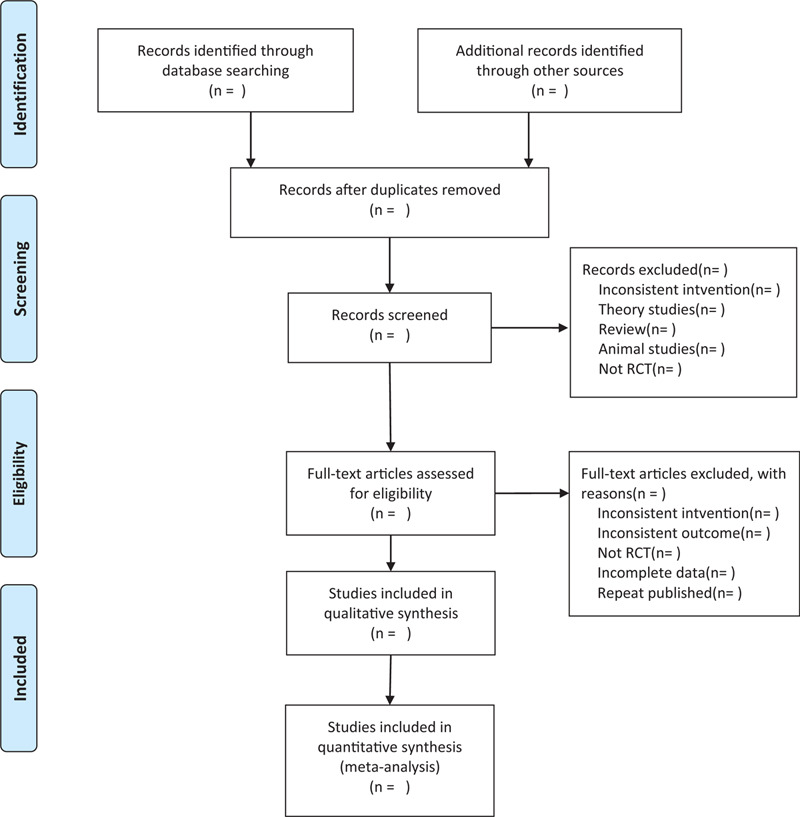
The research flowchart. This figure shows the identification, screening, eligibility, and included when we searching articles.

This review will include the following electronic databases from their inception to Aug. 2020: Electronic database includes PubMed, Embase, Cochrane Library, Web of Science, CNKI (Fig. 1). The research flowchart.

#### 3.6.2 Search strategies

The following MeSH terms and their combinations will be searched:

1. Endometriosis;
2. RCT OR RCTs;
3. Preparation of traditional Chinese medicine OR Chinese herbal compound OR Chinese herbal medicine compound preparation OR Chinese herbal compound prescription OR Traditional medicine compound.

The search strategy for PubMed is shown in (Table 1). Other electronic data bases will be used the same strategy.

**Table 1:**
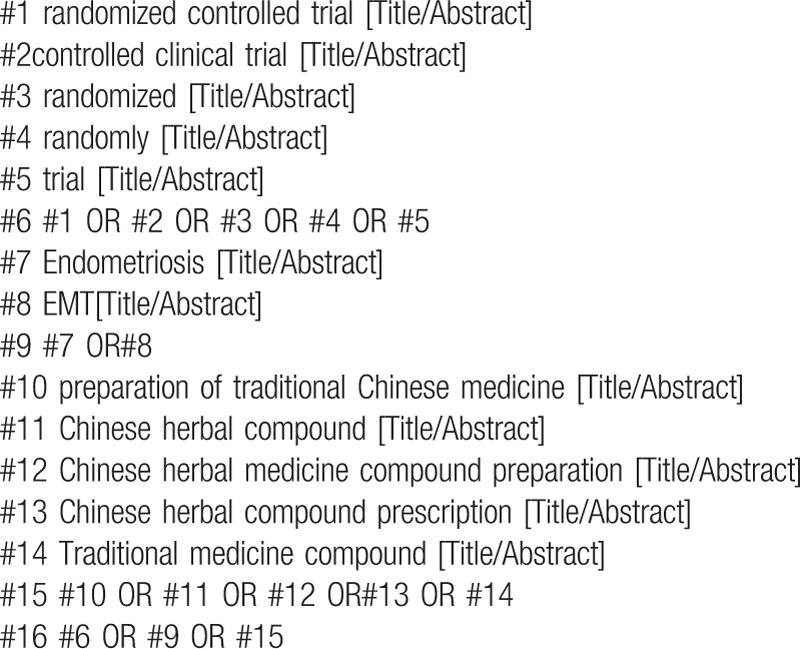
Search strategy in PubMed database.

### 3.7 Data collection and analysis

#### 3.7.1 Studies selection

There will be 2 researchers (XT and JC) carry out the selection of research literature independently using endnote x9 software. We will first make the preliminary selection by screening titles and abstracts. Secondly, we will download full text of the relevant studies for further selection according to the inclusion criteria. If there is any different opinion, 2 researchers will discuss and reach an agreement. If a consensus could not be reached, there will be a third researcher (YL) who make the final decision. The details of selection process will be displayed in the PRISMA flow chart.

#### 3.7.2 Assessment of risk of bias

The assessment of risk of bias will be carried out by 2 independent reviewers (JC and JL), using the Cochrane Collaboration's “Risk of bias” tool. Study bias will be conducted as either: “unclear,” “low,” or “high” risk for the following criteria: random sequence generation, allocation concealment, blinding, incomplete data, selective outcome reporting, and other bias. The assessment of the bias has caused controversy, there is a need for discussion with a third reviewer (SL). The graphic representations of potential bias within and across studies using Rev Man V.5.3.5.

#### 3.7.3 Measures of treatment effect

Statistical analyses will be conducted using the risk ratio with 95% confidence intervals (CIs). Odds ratio (OR) and relative risk (RR) are commonly used for dichotomous outcomes data. For continuous outcomes, the weighted mean difference (WMD) or the standard mean difference (SMD) will be analyzed.

#### 3.7.4 Unit of analysis issues

The unit of analysis will be the individual participant.

#### 3.7.5 Dealing with missing data

Among the results of several studies with insufficient data or missing data, the corresponding author will be contacted to complement the contents. If the corresponding author cannot be contacted, the data alone will be conducted.

#### 3.7.6 Assessment of heterogeneity

The assessment of heterogeneity will be conducted by Review Manager (V.5.3.5). Chi-Squared test and *I*^2^ value of the forest, plot will be calculated to assess heterogeneity, according to the Cochrane Handbook. The *I*^2^ value is classified into 4 levels: little or no heterogeneity (0%–40%), moderate heterogeneity (30%–60%), substantial heterogeneity (50%–90%), and considerable heterogeneity (75%–100%).

#### 3.7.7 Assessment of reporting biases

If the numbers of available studies are sufficient, funnel plots will be assessed reporting biases.

#### 3.7.8 Data synthesis

Review Manager (V.5.3.5) will be used to analyze. The test indicated little or no heterogeneity; a fixed effect model will be used for data. The random effect model will be adopted when there is considerable heterogeneity (*I*^2^ ≥ 50%). If there is considerable variation in results (*I*^2^ ≥ 75%), the meta-analysis will not be performed. The narrative and qualitative summary will be available.

#### 3.7.9 Subgroup analysis and investigation of heterogeneity

Subgroup analysis will be conducted to assess heterogeneity. The different types of Chinese herbal compound prescription (“Shaofuzhuyu decoction”, “Guizhi Fuling Capsule”, “Angelica Sini Decoction”) may be affected heterogeneity.

#### 3.7.10 Sensitivity analysis

Sensitivity analysis will be used to assess the robustness of the results. It is possible to determine according to methodological quality, sample size, and analysis-related issues. The studies that follow a sequence will be removed from all the inclusion reviews. The chi-squared test and *I*^2^ value will be used to quantify statistical heterogeneity.

#### 3.7.11 Summary of evidence

The assessment of evidence for all outcomes will be summarized using the Grading of Recommendations Assessment, Development and Evaluation (GRADE) approach. The quality of evidence will be rated as high, moderate, low, and very low quality.

## 4 Discussion

The main clinical symptoms of EMT include severe pain, dysmenorrhea, difficulty in sexual intercourse, dysuria, infertility, and fatigue.^[9]^ As a major health problem in women of childbearing age, it adversely affect the overall mental health, relationship regulation and overall quality of life of women and their partners.^[10]^ It is not only a common and challenging disease, but also brings a great burden to patients and society, which is related to economic development, social fertility and human development.^[11]^ Importantly, patients with EMT have a high risk of obstetrical complications, such as miscarriage, preterm delivery, preeclampsia, placental abnormalities, etc. In addition, certain acute complications, such as spontaneous peritonitis, may occur in patients with EMT during pregnancy, which is rare but life-threatening.^[12]^ Recent epidemiological studies have shown that, compared with the general population, women with EMT have a significantly higher risk of adverse pregnancy outcome.^[13]^ Therefore, some researchers suggest that continuous health education programs on self-management strategies for women who diagnosed with EMT can improve the quality of life and reduce pain in these patients.^[14]^ For EMT, traditional Chinese medicine compound prescription plays a comprehensive role in the treatment of EMT. The clinical study of traditional Chinese medicine compound prescription shows that it has a good therapeutic effect on patients with EMT.^[15]^

However, the mechanism and standards of treating EMT using Chinese herbal compound prescription are not expounded systematically. In short, this systematic review and meta-analysis help to identify the potential value of Chinese herbal compound prescription in treating EMT and improving the clinical symptoms, related laboratory indexes apparently and life quality. This study provide a foundation for the release of EMT treatment guidelines and treatment options of EMT patients, and thus benefit more patients.

## Author contributions

**Conceptualization:** Xiusong Tang, Jing Chen.

**Data curation:** Jing Chen, Peiqi Ou, Jingqin Chen.

**Formal analysis:** Xiusong Tang, Yehao Luo, Peiqi Ou.

**Funding acquisition:** Yuzhou Pang, Shaohang Lan, Yehao Luo.

**Investigation:** Jing Chen, Peiqi Ou, Jingqin Chen.

**Project administration:** Yuzhou Pang.

**Quality assessment:** Jingqin Chen, Yehao Luo, Jiajing Luo.

**Software:** Jingqin Chen, Jing Chen, Shaohang Lan.

**Supervision:** Yehao Luo, Xiusong Tang.

**Validation:** Yehao Luo, Shaohang Lan.

**Writing – original draft:** Xiusong Tang, Yehao Luo, Jing Chen.

**Writing – review & editing:** Jiajing Luo, Peiqi Ou, Yuzhou Pang.
